# Calnexin is not essential for mammalian rod opsin biogenesis

**Published:** 2008-12-26

**Authors:** Maria Kosmaoglou, Michael E. Cheetham

**Affiliations:** UCL Institute of Ophthalmology, London, UK

## Abstract

**Purpose:**

Misfolding mutations in rod opsin are a major cause of the inherited blindness retinitis pigmentosa. Therefore, understanding the role of molecular chaperones in facilitating rod opsin biogenesis and the response to mutant rod opsin is important for retinal disease and fundamental retinal cell biology. A recent report has shown that *Drosophila* rhodopsin Rh1 requires calnexin (Cnx) for its maturation and correct localization to R1–6 rhabdomeres. In this report, we investigate the role of Cnx in the processing of wild-type and mutant mammalian rod opsin.

**Methods:**

Mouse embryonic fibroblasts (MEFs) from control mice (WT) and mice that express a truncated dysfunctional version of Cnx (sCnx) were used to assess the role of Cnx in the biogenesis, maturation, degradation, and aggregation of mutant and wild-type rod opsin. The mutant P23H rod opsin was used as a prototypical class II misfolding mutant as it is retained in the endoplasmic reticulum (ER) and is either degraded by ER associated degradation (ERAD) or forms aggregates that coalesce to form intracellular inclusions.

**Results:**

Wild-type rod opsin protein translocated normally to the plasma membrane in both cell lines. In contrast, P23H rod opsin was retained in the ER in both cell lines. The only difference observed in rod opsin processing between the WT and sCnx MEFs was a small increase in the incidence of P23H intracellular inclusions in the sCnx cells. This did not appear to be specific for rod opsin, however, as non-rod opsin-expressing sCnx cells also had an increased incidence of ubiquitylated inclusions.

**Conclusions:**

Our data show that, unlike *Drosophila* Rh1, mammalian rod opsin biogenesis does not appear to have an absolute requirement for Cnx. Other chaperones are likely to be more important for mammalian rod opsin biogenesis and quality control.

## Introduction

Rhodopsin, a seven transmembrane domain protein, is a prototypic member of the G-protein coupled receptors (GPCRs) and was the first in this diverse family of proteins to have its crystal structure elucidated [1]. Rhodopsin is formed from the rod opsin protein and the chromophore 11*-*cis*-*retinal. Mutations in rod opsin were first described in 1990 [2] and are the most common cause of autosomal dominant retinitis pigmentosa (ADRP; OMIM 180380). Over 120 point mutations in rod opsin have now been identified (Retnet). Heterologous expression of rod opsin in mammalian cell culture and transgenic animal studies have been used to characterize many of these mutations [3–11]. These studies have revealed two major classes of rod opsin mutations [12]. Class I mutants at the C-terminus of the protein fold normally but are not correctly targeted to the outer segment, whereas class II mutants in the intradiscal and transmembrane domains cause protein misfolding, resulting in retention in the endoplasmic reticulum (ER), degradation, and aggregation. Therefore, it is important to investigate the biogenesis, quality control, and degradation of normal and mutant rod opsin to design therapies for ADRP and enhance our understanding of GPCR biology.

The biogenesis and quality control of multispanning membrane proteins like rod opsin occurs at the ER. Certain steps in this pathway and the potential involvement of molecular chaperones have been discussed elsewhere [13]. The highlights of this process include binding of the rod opsin signal sequence to the signal recognition particle [14] directing the ribosome and the growing polypeptide to the ER membrane. This signal sequence is not cleaved [15], and opsin inserts in the ER cotranslationally [16].

Upon insertion into the ER membrane the N-terminal intradiscal domain of mammalian rod opsin is N-glycosylated at Asn_2_ and Asn_15_ by the oligosaccharyl transferase enzyme [17]. Most glycoproteins use their glycan chains for correct folding and oligomeric assembly [18,19]; however, inhibition of glycosylation by tunicamycin suggests mammalian rod opsin does not require glycan chains for correct folding [6,10]. Furthermore, an intact carbohydrate unit for mammalian rod opsin is not essential for its chromophoric properties or for its regeneration [20]. In contrast, the class II rod opsin mutant P23H requires glycan chains for efficient degradation via endoplasmic reticulum associated degradation (ERAD). Mutant rod opsin accumulation in the ER, observed upon tunicamycin treatment, has revealed a glycan independent quality control mechanism that prevents the mutant protein from escaping the ER [10].

Glycan chains render nascent glycoproteins substrates for resident lectin chaperones of the ER, most notably calnexin (Cnx) and calreticulin [21–23]. This quality control process ensures that only correctly folded, assembled, and modified proteins are transported along the secretory pathway and is a paradigm of protein folding in the ER that includes other folding facilitators such as ERp57, which associates with lectin chaperones Cnx and calreticulin to catalyze glycoprotein disulphide formation/isomerization [24]. The membrane association of a nascent glycoprotein will determine whether it will associate with Cnx, calreticulin, or both [25].

Cnx holds a central role in the folding of many glycoproteins in the ER [22,23,26]. Elegant genetic studies performed with the *Drosophila* homolog of mammalian rod opsin, Rh1, have revealed a requirement for Cnx in its maturation [27]. Mutations in *Drosophila Cnx* led to severe defects in Rh1 expression, whereas other photoreceptor cell proteins were expressed normally, suggesting a specific requirement by Rh1 for this lectin chaperone [27]. *Drosophila* Rh1 has two putative N-glycosylation sites at Asn_20_ and Asn_196_ [28] found on the extracellular domain of the protein [29]. Site-directed mutagenesis of these residues led to the accumulation of Rh1 protein within the ER and retinal degeneration [28–30]. Therefore, there appears to be clear differences between Rh1 and mammalian rod opsin in the requirement for glycosylation during processing. In this study we have investigated if these differences are reflected by a divergence in the need for Cnx in rod opsin biogenesis.

Mice congenitally deficient in the expression of the *Cnx* gene have been previously produced and phenotyped [31]. The homozygous *Cnx*-deficient embryos were carried to full term; however, about 50% died within 2 days after birth and the remainder developed severe motor disorders which led to premature death. A second *Cnx*-deficient mouse strain was generated by an unexpected recombination event, which expressed a truncated Cnx protein (sCnx) resulting from the deletion of a selectable marker [31]. The truncated *Cnx* gene had lost exons 4, 5, and 6, and the protein product was about 15 kDa smaller than the full-length Cnx protein. The deleted region included cysteine residues involved in disulphide bond formation and residues that form the glucose bonding pocket, for binding of monoglucosylated glycoproteins. Unfortunately, the retina was not included in the phenotyping of the *Cnx* null or sCnx mice, and these animals are no longer available for study. Mouse embryonic fibroblasts (MEFs), derived from wild-type (WT) and sCnx mice have been characterized [32]. Replacement of Cnx with sCnx did not affect cell viability or trigger a compensatory upregulation of other ER-resident chaperones such as BiP, PDI, ERp57 or Crt [32]. Importantly, unlike Cnx, sCnx did not associate with newly synthesized glycoproteins; hence, even though sCnx is not a null mutation, and encodes a protein that still targets to the ER, it shows loss of its affinity for glycoproteins, endogenous and transfected, compared to WT MEFs.

Therefore, we used MEFs derived from WT and sCnx mice to test if Cnx was necessary for the biogenesis and maturation of mammalian rod opsin, as it is for *Drosophila* Rh1. The data show that mammalian rod opsin does not appear to require Cnx for normal processing through the secretory pathway or quality control and degradation of mutant rod opsin, but sCnx cells do appear to have general problems with metastable protein folding.

## Methods

WT and sCnx MEFs were a gift of M. Molinari (Institute for Research in Biomedicine, Bellinzona, Switzerland). Lipofectamine and Plus reagent were purchased from Life Technologies (Paisley, UK). 4',6-diamidino-2-phenylindole (DAPI) for nuclear staining, and Protease Inhibitor Cocktail in Dimethyl Sulfoxide (DMSO) for mammalian cell extracts were purchased from Sigma (Dorset, UK). The primary antibodies used were mAb 1D4 to rod opsin, which was a gift from R. Molday (University of British Columbia, Vancouver, Canada). Mouse monoclonal anti-myc antibody (9E10) was from Sigma. Rabbit polyclonal anti-Cnx (SPA-860) was from StressGen Biotechnologies (Cambridge, UK). Goat antimouse antibodies conjugated to horseradish peroxidase were from Pierce (Northumberland, UK). Antimouse secondary Cy3 conjugated secondaries were from Jackson Immunoresearch (Suffolk, UK). The BCA protein assay kit was from Pierce. Bovine wild-type (WT) rod opsin in pMT3 were gifts from D. Oprian (Brandeis University, Waltham, MA). P23H rod opsin in pMT3 was prepared by site-directed mutagenesis using WT pMT3 as a template. WT and P23H constructs were cloned into EcoRI/NotI sites of pMT3 vector and into BamHI/AgeI sites of pEGFP-N1, in frame with GFP, such that the GFP sequence was fused to the C-terminus of rod opsin [10]. His_6_-myc-tagged ubiquitin plasmid was a gift from R. Kopito (Stanford University, Stanford, CA). pEGFPC1 (Clontech, St-Germain-en-Laye, France) was used as a GFP control.

### Cell culture, transfection, and scoring

WT and sCnx MEFs were grown in DMEM/F12 with Glutamax-I+10% (v/v) heat-inactivated fetal bovine serum (FBS) and 1% (v/v) penicillin/streptomycin with an atmosphere of 5% (v/v) CO_2_ at 37 °C. Glass coverslips were treated with Alcian blue dye and were placed in 24 well plates. Glass coverslips were seeded with 5x10^4^ cells per well; 24 h after seeding, the cells were transfected with 0.75 μg DNA per well, with 4 μl Plus, and 2 μl Lipofectamine Plus is a proprietary name for a transfection reagent provided by Life Technologies (Paisley, UK). Next, 24 h after transfection, cells were washed with phosphate buffered saline (PBS, Oxoid; 137 mM sodium chloride, 2.7 mM potassium chloride, 8.1 mM disodium hydrogen phosphate, 1.5 mM potassium dihydrogen phosphate) and were fixed with 4% (v/v) paraformaldehyde (PFA) for 15 min. The slides were mounted with fluorescent mounting medium (DAKO, Cambridgeshire, UK). Transfection efficiency was determined from four independent experiments by scoring the number of rod opsin- (WT and P23H) or GFP-expressing cells as a percentage of the total cells. For morphological analyses, five groups of approximately 100 transfected cells each were counted using a Leica DM RBE Fluorescent microscope in four separate experiments. The distribution of WT opsin-GFP and P23H opsin-GFP in transfected cells was classified either as predominantly plasma membrane, predominantly ER or as containing inclusions. Counts were analyzed using the ANOVA test (ANOVAR), used to compare the means of two samples. Images were collected with a Zeiss LSM 510 laser scanning confocal microscope (Welwyn Garden City, Hertfordshire, UK). The excitation/emission spectrum used for GFP was 488/507. Images were exported from LSM browser to Adobe Photoshop for figure preparation and annotation in Adobe Illustrator (San Jose, CA).

### Immunocytochemistry

Twenty-four hours after transfection, cells were incubated in 3% (v/v) PFA at 37 °C for 10 min and transferred to 0.5% (v/v) PFA at room temperature for 20 min. Cells were incubated in 50 mM NH_4_Cl for 5 min on ice and transferred to PBS at room temperature. Triton X-100 was added at a concentration of 0.5% (v/v) in PBS for 10 min. The slides were washed twice with PBS and blocked for 1 h with PBS containing 10% (v/v) FBS and 10% (v/v) normal donkey serum. Anti-myc (Sigma) antibody (1:1,000) or anti-Cnx (StressGen; 1:600) primary antibody in blocking buffer were added for 1 h. The slides were then washed twice in PBS and donkey antimouse Cy3 (Jackson Immunoresearch) was used at 1:100 in blocking buffer for 1 h. Cells were washed twice with PBS and once with DAPI at a concentration of 2 μg/ml in PBS before mounting in fluorescent mounting medium (DAKO). Fluorescence was observed on a Carl Zeiss LSM 510 confocal laser scanning microscope for image acquisition. The excitation/ emission spectrum used for Cy3 was 543/570 nm. Images were exported from LSM browser to Adobe Photoshop for figure preparation and annotation in Adobe Illustrator.

### Preparation of cell extracts

For 35 mm dishes, the transfection mix containing untagged rod opsin in the pMT3 vector was scaled up 5 times and cells were transfected 24 h after seeding with 4×10^5^ cells per well. Cells were washed twice in ice-cold PBS and incubated in 200 μl PBS/1% n-Dodecyl-β-D-Maltoside (DM) with 1% protease inhibitor cocktail. Cell lysates were centrifuged at 17,500x g for 10 min at 4 °C. Cell fractions were normalized for total protein using the BCA Protein Assay Kit (Pierce) and read with a Safire fluorescence plate reader (Tecan, Ltd., Reading, UK). For SDS–PAGE a volume of 5X modified (30% glycerol) Laemmli sample buffer was added to the soluble fraction. The proteins were resolved on 10% SDS–PAGE gels and were semidry electroblotted (BioRad) onto Protran nitrocellulose membrane (Schleicher & Schuell BioScience, Dassel, Germany). For immunodetection of rod opsin, mAb 1D4 was used at a concentration of 1.33 μg/ml, for GFP anti-av peptide sera (Clontech) was used at 1:2,000, and goat anti-mouse HRP (Pierce) was used at 1:30,000 in PBS+1% (w/v) Marvel (Premier Brands), 0.1% (v/v) Tween-20. The chemiluminescent detection reagent ECL Plus (Amersham Pharmacia Biotech, Little Chalfont, UK) was used to detect immobilized antigens according to the manufacturer’s instructions.

## Results

### Characterization of sCnx cells

**Figure 1:**
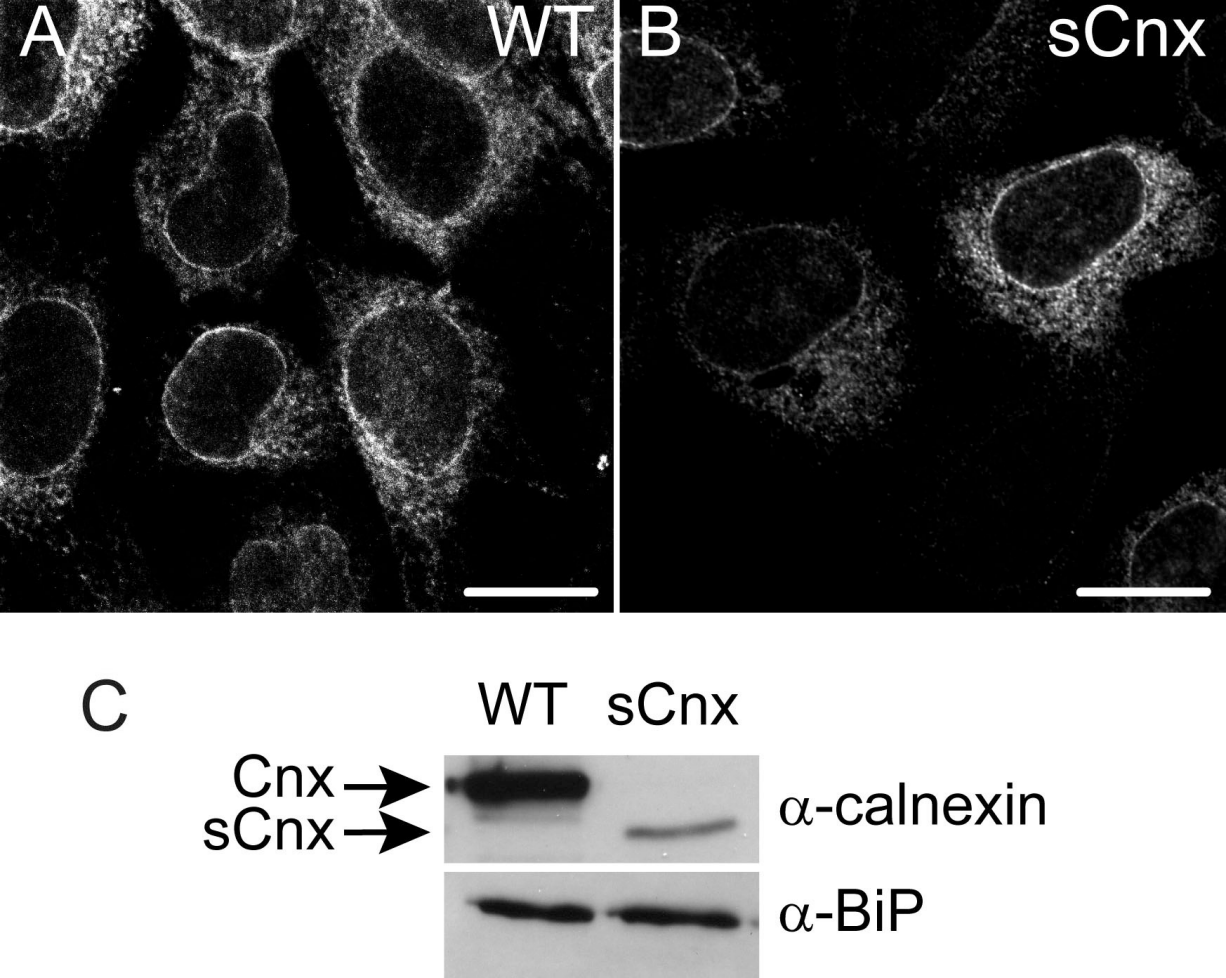
Cnx expression in control and sCnx cells. **A:** Localization of calnexin (Cnx) protein in wild-type (WT) mouse embryonic fibroblasts (MEFs). **B:** Short Cnx localization in sCnx cells. The truncated protein shows the same staining pattern, as it still contains the N-terminal endoplasmic reticulum (ER)-targeting sequence and the C-terminal ER retention motif of full-length Cnx. Note the intensity of the sCnx staining was lower and has been adjusted to reveal the pattern. Scale bar equals 10 μm. **C:** Expression of ER resident chaperones Cnx and BiP in WT and sCnx cells. Western blot revealed reduced expression level and increased electrophoretic mobility of the truncated Cnx protein from sCnx cells and similar BiP levels in both cell lines.

The localization of Cnx in the control WT and sCnx MEFs was investigated by immunocytochemistry. As previously reported [32], both Cnx and sCnx localized to the ER (Figure 1A,B). Importantly, the Cnx immunofluorescence signal for the sCnx cells was lower than for WT cells. Western blotting of detergent extracts of WT and sCnx MEFs confirmed the expression of the truncated Cnx protein in the sCnx cells (Figure 1C). The sCnx protein gives rise to a protein about 15 kDa smaller than the full length Cnx protein. sCnx was present at lower levels than Cnx. Western blotting for BiP confirmed previous data [32] that a compensatory upregulation of other ER resident chaperones had not taken place.

### Wild-type opsin translocates to the plasma membrane in sCnx cells

**Figure 2:**
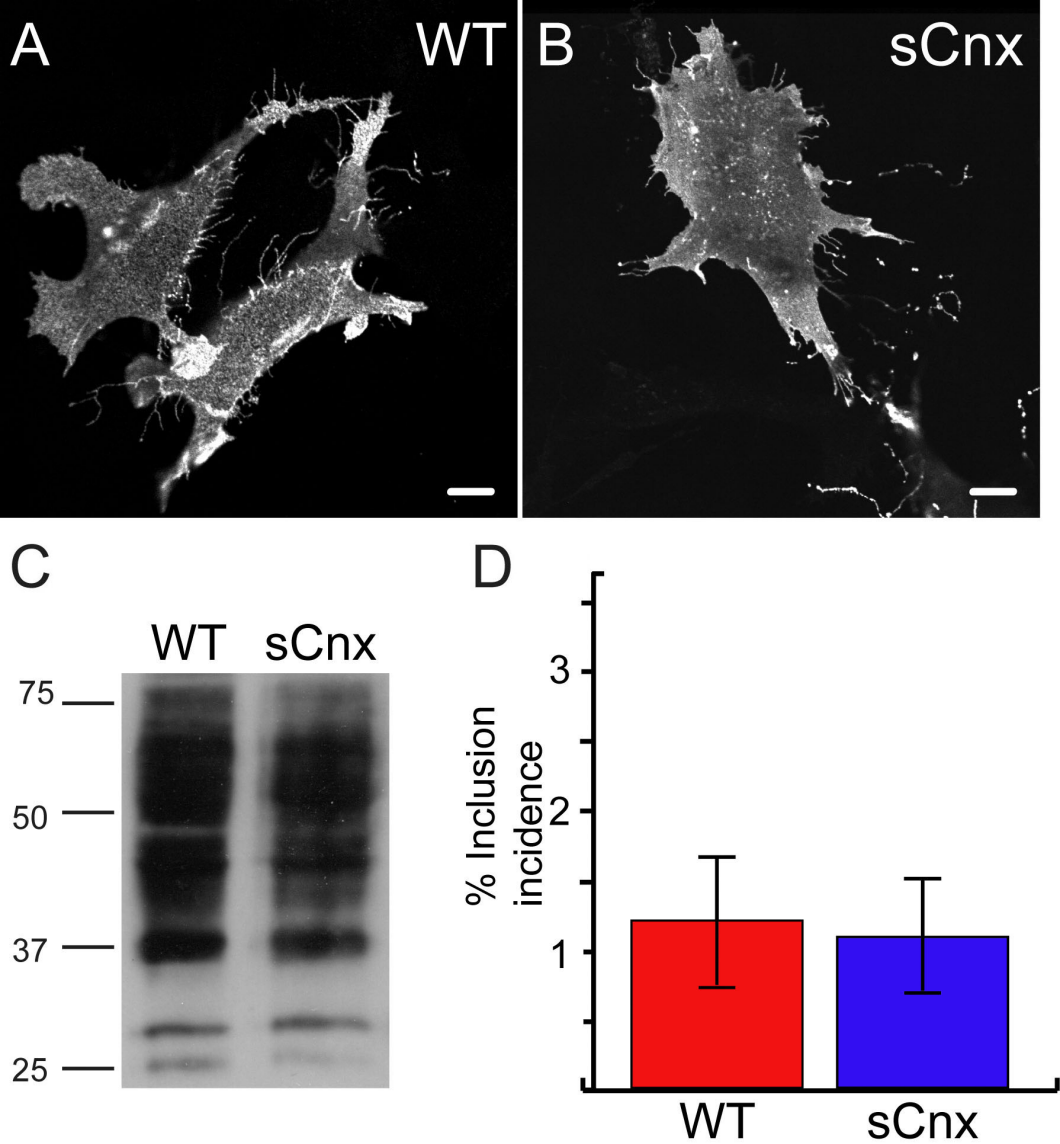
Wild-type rod opsin expression in control and sCnx cells. Wild-type (WT) rod opsin-green fluorescent protein (GFP; WT-GFP) translocates to the plasma membrane of WT (**A**) and calnexin (sCnx) cells (**B**). Scale bar equals 10 μm. **C:** western blotting of untagged rod opsin with mAb 1D4 of 15 μg of soluble protein from WT or sCnx cell lysates (as indicated) revealed no difference in immunoreactive opsin band pattern. This representative blot was selected because of the similar rod opsin expression level between WT and sCnx cells to better demonstrate similar band pattern. The position of molecular weight markers in kDa are indicated on the left. **D**: Quantification of inclusion incidence for WT-opsin-GFP expressing cells after 24 h revealed no significant difference in WT-opsin-GFP aggregation in sCnx cells. Five groups of greater than 100 cells expressing WT-opsin-GFP opsin were scored for the presence of inclusions (the remaining cells had predominantly plasma membrane staining). Error bars represent ±2 Standard Error (SEM). Statistical analysis was performed by ANOVAR followed by posthoc tests. The asterisk indicates p<0.05.

Rod opsin expression has been investigated in a range of cultured cell types. It has been well documented that WT rod opsin translocates to the plasma membrane in the absence of an outer segment [3,6,10]. The localization of WT rod opsin was investigated in WT and sCnx MEFs (Figure 2). WT and sCnx MEFs were transfected with WT rod opsin fused to GFP at the C-terminus (WT-GFP). The localization of rod opsin was investigated by confocal immunofluorescence using the intrinsic GFP fluorescence. In both cell types WT rod opsin was processed to the plasma membrane with no retention in the ER, indicating that the protein had progressed through the secretory pathway (Figure 2A,B). There was no morphological difference in the localization of opsin between the two cell lines, hence Cnx appeared to be dispensable for the correct processing of WT opsin in MEFs. DM detergent soluble cell extracts from WT and sCnx cells transfected with untagged WT rod opsin were analyzed by western blotting and revealed no difference in the 1D4 immunoreactive band pattern (Figure 2C). This confirmed similar processing and glycosylation in WT and sCnx cells. The expression level of WT rod opsin in the sCnx cells, however, was variable and generally lower than in the WT MEFs at equivalent total protein loadings. This corresponded with lower transfection efficiency in the sCnx cells; for example, the transfection efficiency for WT-GFP determined from 4 independent experiments was 27%±11 for the sCnx compared to 44%±22 for the WT MEFs. This did not appear to be rod opsin-specific as similar trends were observed for GFP alone. EndoH and PNGase F digestion of WT rod opsin in soluble cell lysates did not reveal any differences between the WT and sCnx MEFs (data not shown). To test if Cnx may influence the degradation and aggregation of WT rod opsin, WT, and sCnx MEFs were scored for incidence of inclusion formation of WT-GFP. No increase in the small percentage of cells that form WT-GFP inclusions (approximately 1%) was observed in the sCnx cells (Figure 2D).

### P23H rod opsin localizes in the ER and in cytoplasmic inclusions in WT and sCnx cells

**Figure 3:**
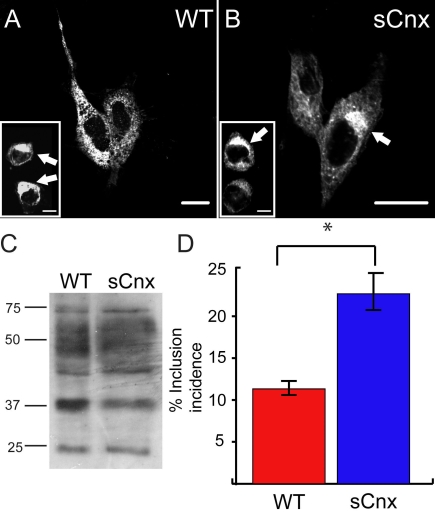
P23H rod opsin expression in WT and sCnx cells. P23H-GFP opsin is retained in the endoplasmic reticulum (ER) and forms intracellular inclusions (arrowed in the inset panel) in wild-type (WT; **A**) and sCnx mouse embryonic fibroblasts (MEFs; **B**). Scale bar equals 10 μm. **C:** western blotting of untagged P23H rod opsin with mAb 1D4 of 15 μg of soluble protein revealed the same glycoform pattern of expression for P23H opsin in WT or sCnx cell lysates (as indicated). This representative blot has been selected for similar rod opsin expression level. The position of molecular weight markers in kDa are indicated on the left. **D:** The incidence of P23H inclusion formation in WT and sCnx MEFs was quantified. Cells were transfected with P23H-opsin-green fluorescent protein (GFP), and the percentages of transfected cells with intracellular inclusions after 24 h were scored blind to experimental status (the noninclusion positive cells had predominant ER staining). Five groups of greater than 100 cells expressing GFP opsin were counted. Error bars represent ±2 SEM. Statistical analysis was performed using ANOVAR followed by posthoc tests. The asterisk indicates p<0.05.

P23H rod opsin has been shown to be retained in the ER before retrotranslocation and degradation by the ubiquitin proteasome system (UPS). If it is not degraded, the misfolded rod opsin can aggregate and is sequestered into cytoplasmic inclusion bodies, resembling aggresomes [9,10]. Similarly here, P23H rod opsin-GFP (P23H-GFP) expressed in both WT and sCnx MEF accumulated in the ER and formed intracellular inclusion bodies (Figure 3A,B). Western analysis revealed the same band pattern for DM soluble untagged P23H rod opsin expressed in both WT and sCnx cells (Figure 3C). EndoH and PNGase F digestion of P23H rod opsin from soluble cell lysates did not reveal any differences between the WT and sCnx MEFs (data not shown). Similar to WT rod opsin, the expression level of P23H rod opsin in the sCnx cells was generally lower than in WT MEFs, corresponding to a lower transfection efficiency (45%±6 for WT MEFs and 28%±13 for sCnx). Rod opsin localization cell counts were generated from 5 fields of roughly 100 cells each. These were scored for ER staining or inclusion formation. Approximately 22% of P23H-GFP expressing sCnx MEFs contained at least 1 inclusion after 24 h, whereas only 11% of P23H-GFP expressing WT MEFs had inclusions at this time (p<0.05) (Figure 3D). This difference may be attributed to the sCnx being unable to process the mutant misfolded P23H opsin as well as the WT cells, but could reflect global protein folding problems in the sCnx cells.

### Enhanced inclusion formation of metastable proteins in sCnx cells

**Figure 4:**
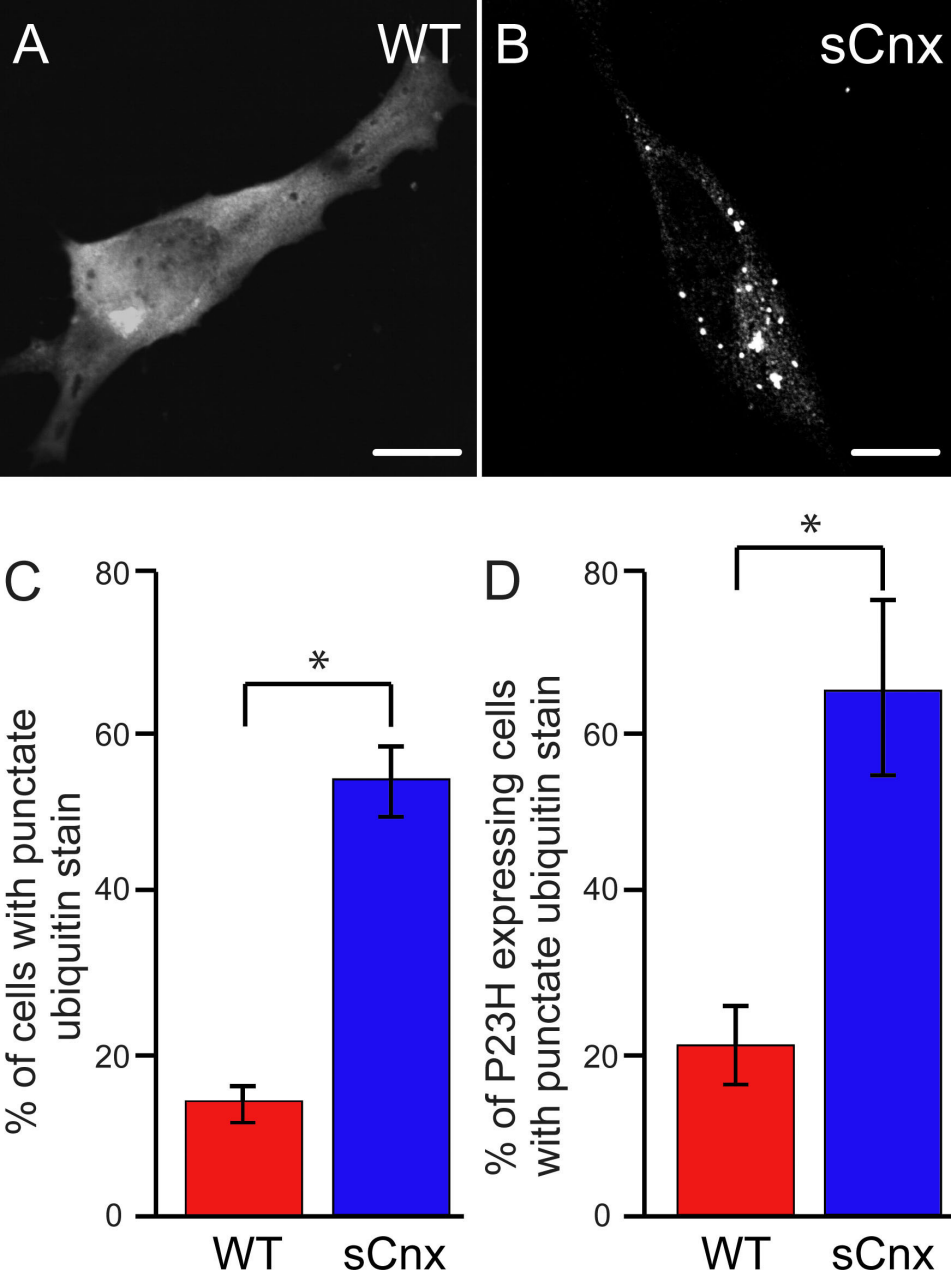
sCnx cells have increased ubiquitylated inclusions. Wild-type (WT; **A**) and calnexin (sCnx; **B**) cells were transfected with a His_6_-myc-tagged ubiquitin expression plasmid and were stained with anti-myc antibody 24 h after transfection. Scale bar equals 10 μm. **C:** Cells were scored for diffuse or punctate ubiquitin stain as exhibited in (**A**) and (**B**). **D:** P23H rod opsin-green fluorescent protein (GFP) and myc-ubiquitin double transfected cells were scored for diffuse or punctate ubiquitin stain. Bar graphs represent an average of three independent experiments. Error bars represent ±2 SEM. Statistical analysis was performed using ANOVAR followed by posthoc tests. The asterisk indicates p<0.05.

To test if the enhanced aggregation observed for P23H rod opsin was specific for mutant rod opsin or reflected more generalized problems of protein folding, we examined the ubiquitin immunoreactivity in WT and sCnx MEFs. Ubiquitin staining can be used to reveal the presence of ubiquitylated inclusions of endogenous unidentified aggregated proteins. WT MEFs transfected with myc-ubiquitin had a diffuse staining pattern characteristic of ubiquitin (Figure 4A). In contrast, a punctate staining pattern characteristic of multiple intracellular inclusions positive for myc-ubiquitin was revealed upon expression in sCnx MEFs, indicating their enhanced stress susceptibility in the folding of endogenous polypeptides (Figure 4B). WT and sCnx MEFs were scored for the presence of these inclusions in 3 independent experiments. WT cells had at least one inclusion in 14% (Figure 4C) of transfected cells while the sCnx MEFs had inclusions in 55% (Figure 4C) of cells (p<0.05). The presence of an aggregation-prone metastable protein in the sCnx cells could be predicted to result in enhanced aggregation because of reduced chaperone activity within these cells. Hence, we expressed P23H rod opsin-GFP with the myc-tagged ubiquitin and scored cells with ubiquitylated inclusions. In the presence of P23H-GFP, the number of ubiquitylated inclusions increased for both WT and sCnx (Figure 4D). The WT cells had at least 1 inclusion in approximately 22% of cells (Figure 4D), whereas the sCnx MEFs had inclusions in 65% (Figure 4D) of cells (p<0.05). The presence of P23H-GFP led to a 8% increase in inclusion incidences in the WT cells and 10% increase in the sCnx cells (p<0.05), suggesting that the aggregation of mutant opsin caused further imbalance in proteostasis, in agreement with previous reports that P23H rod opsin inhibits the UPS [9].

## Discussion

Cnx occupies a central role in the triage of many glycoproteins in the ER [26,33]. Cnx binding retains nascent glycoproteins in the ER to undergo cycles of folding until correct folding is complete and the protein can exit the ER. If Cnx binding is prolonged, then ERAD may be initiated. As *Drosophila* Rh1 has a requirement for Cnx in its maturation [27], we tested the involvement of Cnx in the processing of mammalian rod opsin using a Cnx functionally deficient cell line. We did not observe any differences in the processing of rod opsin in WT and sCnx MEFs. WT rod opsin translocated successfully to the plasma membrane of both cell lines; P23H rod opsin was retained in the ER and formed inclusions in WT as well as sCnx cells. The glycoform pattern of WT and P23H rod opsin observed by western blotting was the same in both cell lines. The only significant difference observed was an increase in inclusion formation for P23H rod opsin in the sCnx MEFs. Therefore, the data clearly support the notion that Cnx is a dispensable chaperone in the processing and ERAD of rod opsin, since the WT and mutant proteins are processed similarly in both cell lines, each targeting its corresponding compartment correctly.

This was not the case for *Drosophila* Rh1, however, which requires Cnx for its maturation [27]. Rh1 in WT flies is localized solely to the rhabdomeres, whereas in Cnx mutant flies Rh1 was detected predominantly in the ER. In a parallel pulse-chase experiment, Rh1 in control flies had matured by 24 h whereas in Cnx mutant flies, Rh1 was initially detected as an immature high MW form that was significantly reduced by 24 h. After 48 h, very little Rh1 was detected, suggesting that most of it had been degraded [27].

*Drosophila* Rh1 and mammalian rod opsin appear to show different requirements for chaperones and chromophore during their biogenesis and quality control. Rh1 was discovered to have a requirement for *NinaA* [34]. Mutant *NinaA^P228^* flies were shown to have a severely reduced Rh1 content in R1–6 rhabdomeres. Subsequently, NinaA was shown to be a specific peptidyl-prolyl-isomerase class chaperone for the maturation of Rh1 [35–37]. Rh1 also requires 3-hydroxyretinal to fold correctly. Lack of 3-hydroxyretinal chromophore either by dietary deprivation or mutations in vitamin A-processing enzymes, such as NinaB dioxygenase and NinaG oxidoreductase, leads to very low levels of detectable Rh1 in rhabdomeres [38]. Previous attempts to express invertebrate visual pigments in mammalian cells were not successful. For example, *Limulus* opsin expressed in COS1 cells was retained within the ER, aggregated, and did not form a functional pigment [39]. This was probably as a result of missing factors in the COS1 cells, such as NinaA and NinaG. These additional requirements for Rh1 processing meant that it was not possible to directly compare the Cnx requirement of Rh1 with mammalian rod opsin expression in sCnx MEFs. However, the reciprocal experiment of expressing bovine rod opsin in *Drosophila* has suggested that a NinaA-type chaperone for mammalian rod opsin may not be required.

Ahmad et al. [40] showed that when bovine rod opsin was expressed in *Drosophila*,it showed stable expression even in the absence of endogenous Rh1 and chromophore and was correctly targeted to *Drosophila* rhabdomeres. Unlike Rh1, the processing of bovine rod opsin was independent of NinaA and NinaG activity, showing a clear difference in the processing of vertebrate rod opsin compared to the endogenous invertebrate rod opsin. The processing was not perfect, however, as the bovine rod opsin from *Drosophila* rhabdomeres exhibited only high mannose oligosaccharides and not the fully mature Golgi forms. Also bovine rod opsin in *Drosophila* did not to couple to Gq although it could successfully activate Gt [40].

According to flybase, *Drosophila* has 3 *Cnx* genes, namely CG9906, Cnx 14D, and Cnx 99A. Rosenbaum et al. [27] identified the cytogenetic locus for the mutant phenotype to 99A7 on *Drosophila* chromosome 3. This locus encodes 4 putative isoforms (A-D). *Drosophila* Cnx99A displays 48.3% amino acid identity with human Cnx, whereas *Drosophila* CG9906 has 50.4% amino acid identity with human Cnx. Therefore, it is not clear which vertebrate form of Cnx would most closely resemble Cnx99A and if this could reflect a specialized invertebrate form of Cnx for Rh1 biogenesis and maturation that is not conserved across species. The Cnx family of molecular chaperones is conserved among plants, fungi, and animals. In mammals there is one major ubiquitous form of Cnx and a tissue-specific form, calmegin, a type-I membrane protein expressed mainly in the spermatids of the testis [41]. In addition to Cnx, calreticulin (Crt) is an ER lumenal homolog. Cnx and Crt associate with a wide array of substrates because they bind monoglucosylated *N*-glycans, which are transiently exposed by glycosylated polypeptides expressed in the ER [33]. However, Cnx and calreticulin were found to associate with distinct sets of polypeptides in cells [42–46]. In addition, the study by Pieren et al. [32] addressed this specifically using WT and sCnx MEFs. The authors found that Crt did not acquire novel substrates upon depletion of Cnx activity. Thus, most cellular Cnx substrates remained inaccessible to Crt even in the absence of Cnx activity [32]. Therefore, we would not anticipate that Crt would compensate for the loss of Cnx activity in these cells or in the retina [41].There is no evidence, as yet, for a mammalian eye-specific form of Cnx.

The increased incidence of P23H rod opsin inclusions in sCnx cells is most likely a consequence of the enhanced stress susceptibility of these cells [47], and not to a specific requirement for Cnx in P23H rod opsin degradation or inclusion clearance. This reduced stress tolerance was also reflected in the lower transfection efficiency and protein expression observed on transient transfection of the sCnx cells with rod opsin or GFP. Cnx occupies a central role in the quality control of glycoproteins either to further folding or degradation. The short nonfunctional version of Cnx expressed in sCnx cells would cause problems for the folding of many nascent glycoproteins in the ER. Therefore, the absence of Cnx activity could affect the stability of metastable proteins within the cells. Alterations in proteostasis, either from increased protein misfolding or impaired chaperone activity, can lead to metastable proteins becoming unstable and revealing their aggregation-prone phenotype. This has been shown to be the case in *C. elegans* temperature-sensitive mutants of muscle paramyosin [48]. In this system, expression of an aggregation-prone polyQ protein was sufficient to cause appearance of the mutant phenotype at the permissive temperature. We observed that the sCnx cells had increased levels of ubiquitylated inclusions both in the presence of transfected P23H rod opsin and in its absence, suggesting that the lack of Cnx and associated imbalance of proteostasis led to the aggregation of metastable proteins. Therefore, the increase in P23H inclusions may not reflect a specific requirement for Cnx, but, instead, it could be a consequence of generalized disturbances of proteostasis in the sCnx cells.

The data highlight that vertebrate rod opsin and Rh1 are different opsins with distinct folding and processing requirements. There are key differences not only in the mechanisms of phototransduction but also in their biogenesis, processing, and photoreceptor organelle. While parallels can be drawn between the two, and *Drosophila* has proved to be an extremely valuable model system, the 2 opsins do not appear to be sufficiently similar to be able to extrapolate findings from one glycoprotein to the other. The mammalian rod photoreceptor encloses a stack of approximately 1,000 flattened disk membranes with 10^4^–10^6^ molecules of rhodopsin per disk. Daily phagocytosis of outer segments by the retinal pigment epithelium leads to the whole outer segment being renewed every 10 days, requiring the vectorial delivery of millions of rhodopsin molecules each day to the base of the outer segment. Rod opsin is synthesized in the ER and further modified in the Golgi. Hence, it is possible that in response to this huge demand, rod opsin biogenesis has become finely tuned and specialized in mammalian rods by the development of dedicated chaperone proteins. The studies of *Drosophila* Rh1 have firmly established the precedent for such dedicated G-protein coupled receptor chaperones, however, the specific chaperone requirements for mammalian rod opsin remain to be identified.

## References

[1] Palczewski K, Kumasaka T, Hori T, Behnke CA, Motoshima H, Fox BA, Le T (2000). I, Teller DC, Okada T, Stenkamp RE, Yamamoto M, Miyano M. Crystal structure of rhodopsin: A G protein-coupled receptor.. Science.

[2] Dryja TP, McGee TL, Reichel E, Hahn LB, Cowley GS, Yandell DW, Sandberg MA, Berson EL (1990). A point mutation of the rhodopsin gene in one form of retinitis pigmentosa.. Nature.

[3] Sung CH, Schneider BG, Agarwal N, Papermaster DS, Nathans J (1991). Functional heterogeneity of mutant rhodopsins responsible for autosomal dominant retinitis pigmentosa.. Proc Natl Acad Sci USA.

[4] Olsson JE, Gordon JW, Pawlyk BS, Roof D, Hayes A, Molday RS, Mukai S, Cowley GS, Berson EL, Dryja TP (1992). Transgenic mice with a rhodopsin mutation (Pro23His): a mouse model of autosomal dominant retinitis pigmentosa.. Neuron.

[5] Sung CH, Davenport CM, Nathans J (1993). Rhodopsin mutations responsible for autosomal dominant retinitis pigmentosa. Clustering of functional classes along the polypeptide chain.. J Biol Chem.

[6] Kaushal S, Ridge KD, Khorana HG (1994). Structure and function in rhodopsin: the role of asparagine-linked glycosylation.. Proc Natl Acad Sci USA.

[7] Sung CH, Makino C, Baylor D, Nathans J (1994). A rhodopsin gene mutation responsible for autosomal dominant retinitis pigmentosa results in a protein that is defective in localization to the photoreceptor outer segment.. J Neurosci.

[8] Li T, Snyder WK, Olsson JE, Dryja TP (1996). Transgenic mice carrying the dominant rhodopsin mutation P347S: evidence for defective vectorial transport of rhodopsin to the outer segments.. Proc Natl Acad Sci USA.

[9] Illing ME, Rajan RS, Bence NF, Kopito RR (2002). A rhodopsin mutant linked to autosomal dominant retinitis pigmentosa is prone to aggregate and interacts with the ubiquitin proteasome system.. J Biol Chem.

[10] Saliba RS, Munro PM, Luthert PJ, Cheetham ME (2002). The cellular fate of mutant rhodopsin: quality control, degradation and aggresome formation.. J Cell Sci.

[11] Tam BM, Moritz OL (2006). Characterization of rhodopsin P23H-induced retinal degeneration in a Xenopus laevis model of retinitis pigmentosa.. Invest Ophthalmol Vis Sci.

[12] Mendes HF (2005). van der SJ, Chapple JP, Cheetham ME. Mechanisms of cell death in rhodopsin retinitis pigmentosa: implications for therapy.. Trends Mol Med.

[13] Kosmaoglou M, Schwarz N, Bett JS, Cheetham ME. Molecular chaperones and photoreceptor function. Prog Retin Eye Res. 2008. 10.1016/j.preteyeres.2008.03.001 PMC2568879 18490186

[14] Audigier Y, Friedlander M, Blobel G (1987). Multiple topogenic sequences in bovine opsin.. Proc Natl Acad Sci USA.

[15] Friedlander M, Blobel G (1985). Bovine opsin has more than one signal sequence.. Nature.

[16] Kanner EM, Klein IK, Friedlander M, Simon SM (2002). The N-terminus of opsin translocates “posttranslationally” as efficiently as cotranslationally.. Biochemistry.

[17] Kean EL (1999). The dolichol pathway in the retina and its involvement in the glycosylation of rhodopsin.. Biochim Biophys Acta.

[18] Land A, Braakman I (2001). Folding of the human immunodeficiency virus type 1 envelope glycoprotein in the endoplasmic reticulum.. Biochimie.

[19] Paulsson K, Wang P (2003). Chaperones and folding of MHC class I molecules in the endoplasmic reticulum.. Biochim Biophys Acta.

[20] Renthal R, Steinemann A, Stryer L (1973). The carbohydrate moiety of rhodopsin: lectin-binding, chemical modification and fluorescence studies.. Exp Eye Res.

[21] Hebert DN, Foellmer B, Helenius A (1995). Glucose trimming and reglucosylation determine glycoprotein association with calnexin in the endoplasmic reticulum.. Cell.

[22] Hebert DN, Molinari M (2007). In and out of the ER: protein folding, quality control, degradation, and related human diseases.. Physiol Rev.

[23] Molinari M (2007). N-glycan structure dictates extension of protein folding or onset of disposal.. Nat Chem Biol.

[24] Caramelo JJ, Parodi AJ (2008). Getting in and out from calnexin/calreticulin cycles.. J Biol Chem.

[25] Helenius A, Aebi M (2004). Roles of N-linked glycans in the endoplasmic reticulum.. Annu Rev Biochem.

[26] Moremen KW, Molinari M (2006). N-linked glycan recognition and processing: the molecular basis of endoplasmic reticulum quality control.. Curr Opin Struct Biol.

[27] Rosenbaum EE, Hardie RC, Colley NJ (2006). Calnexin is essential for rhodopsin maturation, Ca^2+^ regulation, and photoreceptor cell survival.. Neuron.

[28] Katanosaka K, Tokunaga F, Kawamura S, Ozaki K (1998). N-linked glycosylation of Drosophila rhodopsin occurs exclusively in the N-terminal domain and functions in rhodopsin maturation.. FEBS Lett.

[29] Webel R, Menon I, O'Tousa JE, Colley NJ (2000). Role of asparagine-linked oligosaccharides in rhodopsin maturation and association with its molecular chaperone, NinaA.. J Biol Chem.

[30] O'Tousa JE (1992). Requirement of N-linked glycosylation site in Drosophila rhodopsin.. Vis Neurosci.

[31] Denzel A, Molinari M, Trigueros C, Martin JE, Velmurgan S, Brown S, Stamp G, Owen MJ (2002). Early postnatal death and motor disorders in mice congenitally deficient in calnexin expression.. Mol Cell Biol.

[32] Pieren M, Galli C, Denzel A, Molinari M (2005). The use of calnexin and calreticulin by cellular and viral glycoproteins.. J Biol Chem.

[33] Ellgaard L, Frickel EM (2003). Calnexin, calreticulin, and ERp57: teammates in glycoprotein folding.. Cell Biochem Biophys.

[34] Larrivee DC, Conrad SK, Stephenson RS, Pak WL (1981). Mutation that selectively affects rhodopsin concentration in the peripheral photoreceptors of Drosophila melanogaster.. J Gen Physiol.

[35] Colley NJ, Baker EK, Stamnes MA, Zuker CS (1991). The cyclophilin homolog ninaA is required in the secretory pathway.. Cell.

[36] Stamnes MA, Shieh BH, Chuman L, Harris GL, Zuker CS (1991). The cyclophilin homolog ninaA is a tissue-specific integral membrane protein required for the proper synthesis of a subset of Drosophila rhodopsins.. Cell.

[37] Baker EK, Colley NJ, Zuker CS (1994). The cyclophilin homolog NinaA functions as a chaperone, forming a stable complex in vivo with its protein target rhodopsin.. EMBO J.

[38] Ahmad ST, Joyce MV, Boggess B, O'Tousa JE (2006). The role of Drosophila ninaG oxidoreductase in visual pigment chromophore biogenesis.. J Biol Chem.

[39] Knox BE, Salcedo E, Mathiesz K, Schaefer J, Chou WH, Chadwell LV, Smith WC, Britt SG, Barlow RB (2003). Heterologous expression of limulus rhodopsin.. J Biol Chem.

[40] Ahmad ST, Natochin M, Barren B, Artemyev NO, O'Tousa JE (2006). Heterologous expression of bovine rhodopsin in Drosophila photoreceptor cells.. Invest Ophthalmol Vis Sci.

[41] Watanabe D, Yamada K, Nishina Y, Tajima Y, Koshimizu U, Nagata A, Nishimune Y (1994). Molecular cloning of a novel Ca(2+)-binding protein (calmegin) specifically expressed during male meiotic germ cell development.. J Biol Chem.

[42] van Leeuwen JE, Kearse KP (1996). The related molecular chaperones calnexin and calreticulin differentially associate with nascent T cell antigen receptor proteins within the endoplasmic reticulum.. J Biol Chem.

[43] Halaban R, Cheng E, Zhang Y, Moellmann G, Hanlon D, Michalak M, Setaluri V, Hebert DN (1997). Aberrant retention of tyrosinase in the endoplasmic reticulum mediates accelerated degradation of the enzyme and contributes to the dedifferentiated phenotype of amelanotic melanoma cells.. Proc Natl Acad Sci USA.

[44] Pipe SW, Morris JA, Shah J, Kaufman RJ (1998). Differential interaction of coagulation factor VIII and factor V with protein chaperones calnexin and calreticulin.. J Biol Chem.

[45] Danilczyk UG, Cohen-Doyle MF, Williams DB (2000). Functional relationship between calreticulin, calnexin, and the endoplasmic reticulum luminal domain of calnexin.. J Biol Chem.

[46] Molinari M, Eriksson KK, Calanca V, Galli C, Cresswell P, Michalak M, Helenius A (2004). Contrasting functions of calreticulin and calnexin in glycoprotein folding and ER quality control.. Mol Cell.

[47] Coe H, Bedard K, Groenendyk J, Jung J, Michalak M. Endoplasmic reticulum stress in the absence of calnexin. Cell Stress Chaperones. 2008. 10.1007/s12192-008-0049-x PMC2673926 18528784

[48] Gidalevitz T, Ben-Zvi A, Ho KH, Brignull HR, Morimoto RI (2006). Progressive disruption of cellular protein folding in models of polyglutamine diseases.. Science.

